# Letting go or giving up? The influence of self-transcendence meaning of life on goal adjustment in high action crisis

**DOI:** 10.3389/fpsyg.2023.1054873

**Published:** 2023-02-01

**Authors:** Xinyi Hu, Heyi Zhang, Meifang Geng

**Affiliations:** ^1^The Psychological Quality Education Centre, Beijing Jiaotong University, Beijing, China; ^2^Faculty of Education, Beijing Normal University, Beijing, China

**Keywords:** self-transcendence meaning of life, action crisis, goal disengagement, goal reengagement, perceived self-efficacy

## Abstract

When individuals pursuing personal goals encounter setbacks and failures, they often fall into a conflict between disengaging from and striving toward the goal, defined as an action crisis. The present study investigated the influence and mechanism of self-transcendence meaning of life (STML) on goal disengagement and reengagement during a high versus a low action crisis. Study 1 included situations with different action crises. In Study 1, participants with high STML exhibited significantly higher goal disengagement and reengagement during high action crisis compared with low action crisis. Study 2 replicated the findings in Study 1 using participants’ personal goals by questionnaires, and further exhibited that action crisis had negative effect on self-efficacy for participants with low STML, and this process subsequently reduced goal adjustment. Interestingly, no mediation effect of self-efficacy was found between action crisis and goal adjustment among participants with high STML. Findings from the present study suggest that releasing obsessions and adopting a dialectical relationship between success and failure may help individuals in high action-crisis situations, and self-efficacy may provide flexibility and autonomy.

## 1. Introduction

Personal goals are psychological representations of desired outcomes that provide value and meaning to life. Individuals use a process of life crafting to make their goals congruent with their values and wishes (Schippers and Ziegler, 2019), and this process can bring meaning and fulfillment. Further, goal realization is a source of self-identity and well-being that leads to successful development (Haase et al., 2021). However, goal realization is not always possible when individuals encounter challenging obstacles. Coping with ongoing obstacles may prove ineffective or counterproductive to the goal itself. Additionally, continued commitment to certain goals without successful coping strategies may even lead to poor mental health (Wrosch and Scheier, 2020). Goal disengagement, or the removal of a commitment to previously anticipated but unattainable goals, often occurs if striving toward an ineffective goal is too costly (Wrosch et al., 2003; Heckhausen et al., 2019). Goal disengagement may help individuals discontinue meaningless pursuits. Furthermore, goal reengagement refers to engaging in new goals in the presence of unattainable goals (Haase et al., 2021). Goal reengagement is the practice of redirecting commitments to meaningful life goals and may help individuals find self-worth (Barlow et al., 2019; Brandstätter and Bernecker, 2022). Disengagement and reengagement of goals are both adaptive decision-making processes that can help individuals maintain mental and physical health (Wrosch et al., 2007; Brassen et al., 2012).

Although these two facets of goal adjustment can contribute to adaptive development, decision-making may be difficult for many individuals, particularly when the decision is associated with a long-term goal. Despite hard work, some failures are inevitable and place individuals in a state of action crisis (Brandstätter and Schüler, 2013), conceptualized as a dynamic and dilemmatic time during which individuals challenge their goals, deliberate costs and profits of committing to the goal, and determine whether to persist or quit (Ghassemi et al., 2017). For example, when research is difficult to continue and the family is in immediate financial need, a graduate student might have to decide whether to continue studying or drop out, thus facing an action crisis. In an action crisis, individuals find that effort is no longer useful and tend to refocus from goal-related performance to expectancy-and value-related information (Brandstätter and Schüler, 2013). During an action crisis, the goal’s original attainability and desirability decreases, as do any positive emotions associated with the goal, while negative affect and doubt increase (Ghassemi et al., 2021). Indeed, past research suggests that action crisis reduces psychological and physiological well-being (Brandstätter et al., 2013). However, states of action crisis may also have a positive impact, as it may provide the chance to consider goal worthiness and evaluate potential new goals (Wrosch et al., 2003). An action crisis typically occurs prior to goal disengagement (Herrmann and Brandstätter, 2015), which promotes attainability to alternative goals (Brandstätter and Herrmann, 2016). Therefore, making individual goal adjustment choices, particularly goal disengagement and reengagement, may be beneficial when facing an action crisis. The present study explored the influence and mechanism of self-transcendence meaning of life on goal disengagement and reengagement under high action crisis, aiming to test the protective effects of wisdom from Eastern philosophy in an action crisis.

## 2. Theories and hypotheses

### 2.1. Self-transcendence meaning of life as a factor associated with goal adjustment in high action crisis

Self-transcendence meaning of life (STML) refers to beliefs that are transcendent to one’s self (versus self-centered beliefs) and present as a transcendental or detached attitude toward life (Li, 2006). STML has two core traits that are rooted in Eastern cultures. One trait is based on Buddhism’s “remove thought of focus on self” (Li, 2006), which refers to removing attachment to the self and reducing concern for the self; the rationale is that, in Buddhist philosophy, self-obsession is the root of all pain and suffering (Wang and Jing, 2017). Therefore, removing self-obsession refers to an attitude that is detached from success or failure and seeks to abandon obsessiveness. The other trait is giving meaning to loss and accepting (or resigning oneself to accept) unpleasant facts or results, which comes from Taoism. In Taoist philosophy, everything in the world is dialectical and meaningful, thus, gain and loss, and success or failure are also dialectical and meaningful and can therefore be accepted (Li, 2006).

Prior studies suggest that STML may be a protective factor during high action crises. First, past studies have found that higher STML leads to increased well-being and decreased depression and anxiety (Wang and Jing, 2017), while lower STML is associated with increased depression and anxiety symptoms among college students in high stress situations (Li, 2006). Thus, STML may be protective from high stress during high action crises. Findings from prior studies suggest that STML might help individuals choose goal disengagement as opposed to maintaining an unattainable goal during a high action crisis. When facing an action crisis, individuals are easily immersed in rumination (Brandstätter and Schüler, 2013). STML can help individuals get outside of themselves and connect with something larger, rather than focus on their own experiences from a psychologically immersed perspective (Reischer et al., 2021). STML can also help individuals maintain a detached attitude or suppress the pursuit of an unachievable goal in an action crisis. Further, when individuals are impacted by an action crisis and consider disengaging from a goal, it may result in withdrawn effort and commitment prior to admitting defeat. Disengaging goals in high action crisis may be seen as a failure; however, as noted above, STML can help people accept failure and encourage them to see meaning in failure (Li, 2006). Therefore, individuals with high STML might peacefully disengage from unattainable goals without becoming defensive; such individuals are not afraid of losing or of failure. Finally, during an action crisis, individuals tend to distance themselves from their goals and consider the desirability and attainability of the goal during high action crisis (Ghassemi et al., 2017). Individuals may also devalue goal-relevant resources in more practical ways (Herrmann et al., 2019). For instance, maintaining an unbiased perspective may be essential and necessary so that the dialectical and detached aspects of STML may be used to promote goal disengagement. Therefore, STML may promote goal disengagement during action crisis.

STML may also influence goal reengagement. For instance, during a high action crisis, if alternative options for goal pursuit are scarce, disengaging from the goal is not adaptive (O'Connor et al., 2009). While the factors that influence goal reengagement remain unknown, Haase et al. (2021) described specific aspects of well-being (including positive emotion and life satisfaction) that increase goal reengagement capacity. As previously stated, STML can have positive effect on well-being (Wang and Jing, 2017; Papaioannou and Krommidas, 2021). Given that STML is positively associated with well-being, STML may also be beneficial to goal reengagement. In addition, according to the self-concordance model of goal striving (Sheldon and Elliot, 1999), pursuing self-concordant goals may promote positive mental health and well-being. Previous studies have found that individuals with high self-transcendence describe their experiences of life as spiritual journeys of humanistic growth (Reischer et al., 2021), thus, people with high STML may be more likely to choose self-concordant goals that focus on core values and dynamic interests rather than goals that focus on rewards and achievement. This internal motivation process may help individuals exhibit higher goal reengagement desire during high action crisis.

Based on the abovementioned reasons, goal adjustment capacities, namely goal disengagement and reengagement, with individual differences may appear during action crisis. We propose that STML may be a protective factor for individuals in high action crisis. Therefore, we hypothesized that participants with high STML would exhibit higher goal disengagement and reengagement during high action crisis compared with low action crisis (hypothesis 1).

### 2.2. The mediating effect of perceived self-efficacy

Self-efficacy is defined as a general belief in overall competence across different achievement situations (Chen et al., 2001). Individual differences of task capability can inform perceived self-efficacy (Vohs et al., 2013). The relationship between action crisis and STML on goal adjustment may be mediated by self-efficacy.

Prior studies have found that self-efficacy is challenged and unstable during crisis situations (Park and Avery, 2019). Action crisis has also been found to be negatively associated with general self-efficacy (Wolf et al., 2018). Therefore, high action crisis may be associated with low self-efficacy perception. However, those with higher STML may be an exception. Previous studies have found that spiritual well-being is related to self-efficacy (Hasanshahi and Mazaheri, 2016), and that adolescents’ academic self-efficacy is associated with self-transcendence (Metilda and Maheswari, 2017). Once individuals view themselves in a dialectical, expansive, and non-self-centered view, a new sense of identity is formed (Eaude, 2009), and it may promote understanding and acceptance by expanding self-boundaries (Ko et al., 2017). Therefore, those with lower STML may exhibit lower perceived self-efficacy during high action crisis, while participants with higher STML may exhibit stable self-efficacy during either high or low action crisis.

Self-efficacy may be positively associated with goal adjustment. Bandura (1997) suggested that self-efficacy may influence goal choice and goal revision. General self-efficacy may help individuals successfully adapt to new and adverse situations (Pulakos et al., 2000). Self-efficacy is positively correlated with autonomous motivation, suggesting that individuals with high self-efficacy may be autonomously motivated to choose appropriate goals based on their interests and personal importance (Wolf et al., 2018). Those with an acquired brain injury with higher self-efficacy may attain important life goals successfully, which promotes higher life quality (Brands et al., 2014). The above results indicate that self-efficacy may have a positive effect on goal-level change processes, including choice or revision. Self-efficacy may also help determine the attribution of attainments and failures (Pajares, 2002). Self-efficacy protects an individual’s self-esteem by providing both uncontrollable and flexible attributions, which may help to reduce defenses, accept reality, and disengage from unattainable goals. Self-efficacy also aids in individualistic goal choice, as high self-efficacy provides a wider array of realistic and valuable goals (Bandura, 2012). Thus, those with higher self-efficacy may have more autonomy to choose goal disengagement and reengagement.

**Figure 1:**
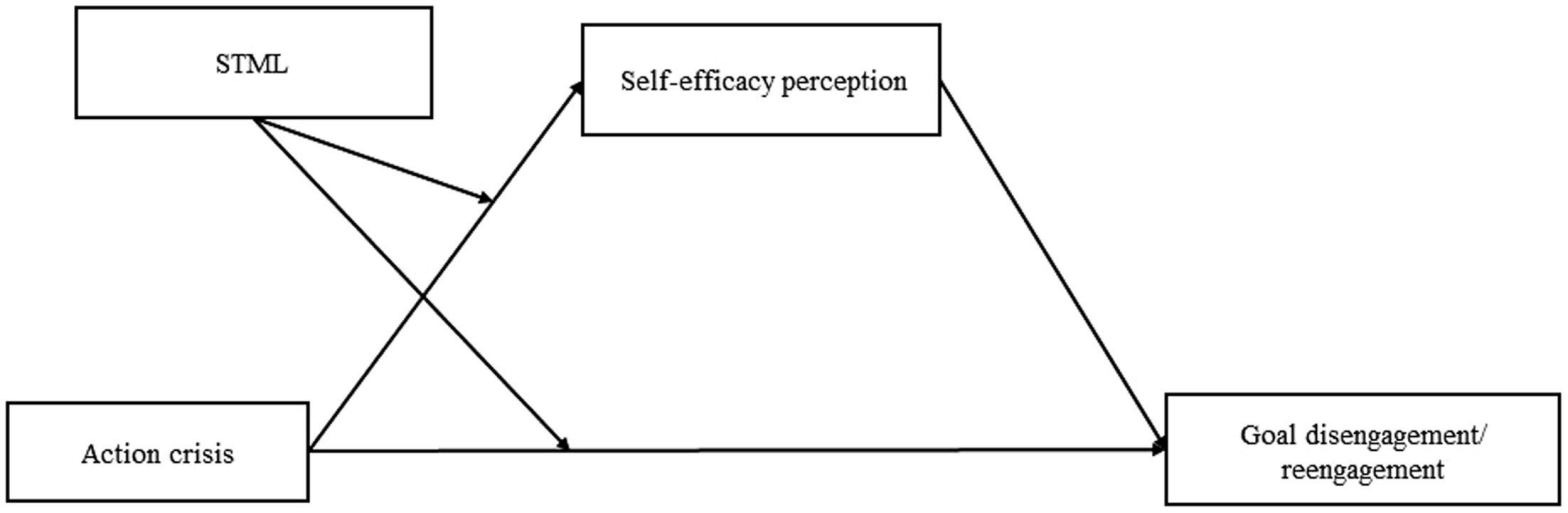
Conceptual models for the analysis.

Based on the abovementioned findings, self-efficacy may function as a mediator in the association between action crisis and goal adjustment among participants with low STML. Further, we hypothesized that individuals in high action crisis with lower STML may exhibit lower self-efficacy, and subsequently, a reduced capability to disengage and reengage from goals. Among individuals with higher STML, we hypothesized that their self-efficacy would not be influenced by action crisis (hypothesis 2, a moderated mediation hypothesis; see Figure 1).

## 3. Study 1

The aim of Study 1 was to examine the association between action crisis and goal adjustment (namely goal disengagement and reengagement) under high versus low STML. We hypothesized that for participants with high STML (versus low STML), high action crises would result in high goal disengagement and reengagement (hypothesis 1).

### 3.1. Method

#### 3.1.1. Participants and procedure

Two hundred and five participants were recruited from a university located in Beijing. All participants were students who enrolled in an elective psychology course, and they received course credit for their participation. Inclusion criteria were as follows: (1) volunteered to participant; (2) finished all questionnaire items. The participants who chose one response option for all items or chose response items in a pattern were also excluded, as this response style may suggest that participants were not answering the items conscientiously. After participant exclusions following the above criteria, a total of 188 participants (109 males) with a mean age of 20.46 (range 17–26, *SD* = 1.75) were included in Study 1 analyses.

Participants completed online questionnaires. They were informed that their personal information would remain confidential, and they could quit at any time during the task. First, participants completed the Self-Transcendence Meaning of Life Scale. Second, they were randomly assigned to high or low action crisis condition. We generated a series of random digits containing the numerals 1 and 2 in advance and distributed the numbers to the participants according to the order of their student ID. Thus, participants completed different versions of online questionnaires containing different scenarios according to their randomized number (1 refers to low action crisis, and 2 refers to high action crisis). Participants were then instructed to read the scenarios containing different levels of action crisis (see details in *Action crisis manipulation*) and complete the goal disengagement and goal reengagement scale according to the scenario. Finally, participants answered questions for a manipulation check. To check if the action crisis manipulation was effective, participants completed four items from the Action Crisis Scale according to the goals mentioned in the manipulation story. Participants also completed two items to determine whether they identified with the character in the story (see details in *Manipulation check*).

#### 3.1.2. Measures

##### 3.1.2.1. Self-transcendence meaning of life

The Chinese version of the Self-Transcendence Meaning of Life (STML) Scale (Li, 2006) was used to assess self-transcendence life meaning (i.e., positive views and attitudes about gain, loss, success, and failure in life). The STML Scale contains two dimensions: obtain the meaning of failure (three items; e.g., “Loss may be more meaningful than gain in life”; “Loss always teaches people much more”; Cronbach’s *α* = 0.72.) and detachment from success or failure (five items; e.g., “Life lies not in gain and loss but in enrichment”; “Both success and failure are positive for people”; Cronbach’s *α* = 0.85). The items were rated on a 4-point scale (1 = strongly disagree; 4 = strongly agree). Higher scores indicated higher STML. Previous studies demonstrated that the scale had good validity and reliability in China (Wang and Jing, 2017). Cronbach’s *α* for the scale was 0.86 in the present study.

##### 3.1.2.2. Action crisis manipulation

Participants were randomly assigned to high or low action crisis: 94 participants were assigned to high action crisis, and the other 94 were assigned to low action crisis. Participants read one of two different scenarios depending on group assignment, similar to prior work (Brandstätter and Schüler, 2013). Participants were asked to imagine that they were the protagonist of the scenarios. The scenario read by the high action crisis group was as follows:

Zhang is about to graduate from college. Four years ago, Zhang’s goal was to find a job and raise a family in a big city. However, Zhang finds it difficult to get a good job in the big city due to intense competition. Zhang also finds that house prices and the general cost of living in the city is very high, and it is too crowded for his/her taste.

The scenario read by the low action crisis group was as follows:

Zhang is about to graduate from college. Four years ago, Zhang’s goal was to live in a small and beautiful city. Zhang finds a small city close to his/her hometown with acceptable housing prices, and easily finds a suitable job. The competition in the city was not intensive at all.

##### 3.1.2.3. Goal disengagement and goal reengagement

The Goal Disengagement Scale and Goal Reengagement Scale (Wrosch et al., 2003) were used to measure participants’ attitudes toward Zhang’s experience in the story. These scales were translated from English following a standard back-translation procedure, and were presented in Chinese. The subjects of the sentences were changed from “I” to “Zhang,” and the statements were adjusted because the items were based on participants’ conjectures about the protagonist in the scenarios. The Goal Disengagement Scale contains four items (e.g., “*I think Zhang will find it difficult to stop trying to achieve the goal*,” reverse scored); the Goal Reengagement Scale contains six items (e.g., “*I think Zhang will seek other meaningful goals*”). Instructions for the two scales were as follows: “In the story, Zhang’s goal has not yet been achieved. What do you think Zhang will do since his/her goal is not achieved now? Please choose the options that mostly fit with your attitudes about each item.” The items were rated on a 5-point scale (1 = strongly disagree, 5 = strongly agree). Higher scores indicated higher goal disengagement or reengagement. Previous studies demonstrated the scale had good validity and reliability (Haase et al., 2021). In the present study, Cronbach’s *α* was 0.73 for goal disengagement and 0.89 for goal reengagement.

##### 3.1.2.4. Manipulation check

To determine whether different levels of action crisis were implemented, participants completed four items from the Action Crisis Scale (Brandstätter and Schüler, 2013). The subjects in the item sentences were also changed from “I” to “Zhang” (e.g., “*Zhang would repeatedly ruminate about this goal*”). The scale was translated from English following a standard back-translation procedure. The items were rated on a 5-point scale (1 = strongly disagree, 5 = strongly agree). Higher scores reflect higher action crisis. The other two items we did not use in Study 1 were “I repeatedly have not done anything for the specific goal despite the intention to do so,” and “When pursuing my goal, I am repeatedly confronted with situations where I do not know how to continue.” The above two items were abandoned because the participants needed to surmise the attitude of protagonist (Zhang) in the special scenarios, and we thought the participants would have too much difficulty surmising these two items as spectators based on the limited information. Previous studies showed that the scale had good validity and reliability (Ghassemi et al., 2017). In the present study, Cronbach’s *α* of the four items was 0.72.

Participants were expected to identify with the protagonist in the story, since this was considered as a necessary condition for them to consider the goal disengagement and reengagement described in the story. Therefore, participants were asked to estimate how strongly they identified with Zhang by answering the following two questions (Brandstätter and Schüler, 2013): “*How much do you identify with Zhang in the above story?*,” and “*Were you imagining yourself completely as Zhang in the story?*” The items were rated on a 7-point scale (1 = not at all;7 = very much). The two abovementioned items were significantly correlated (*r* = 0.83, *p* < 0.001). The two items were then averaged as an indicator of participants’ identification with the protagonist, and higher scores reflected higher identification.

#### 3.1.3. Data analysis

First, SPSS 22.0 was used to test the manipulation check. Second, the computational tool PROCESS (version 2.16, Hayes, 2013) was used to test the moderation effect. The scale-level variables were mean centered before analysis. Bootstrap analyses were based on 5,000 bootstrap samples, and 95% confidence intervals (95% CIs) were used to determine whether the moderation effect was significant. When the confidence interval for the interaction did not contain 0, the effect was significant.

### 3.2. Results and discussion

Participants assigned to read the high action crisis story exhibited a significantly higher action crisis score (*M* = 3.97, *SD* = 0.60) compared with participants assigned to read the low action crisis story (*M* = 2.87, *SD* = 0.67), *t*(186) = −11.89, *p* < 0.001. The two items used to determine whether participants identified with the protagonist were averaged, thus, scores ranged from 1 to 7. We examined the difference between the identification scores and 4 (mid-point of the scale). Participants’ identification scores were higher than the mid-point of the scale, *M* = 4.37, *SD* = 1.55, *t*(187) = 3.24, *p* = 0.001. The identification scores between high (*M* = 4.35, *SD* = 1.68) and low action crisis (*M* = 4.39, *SD* = 1.42) groups were not significant, *t*(186) = 0.18, *p* = 0.85. Goal disengagement and reengagement were significantly correlated (*r* = 0.25, *p* < 0.001).

**Figure 2:**
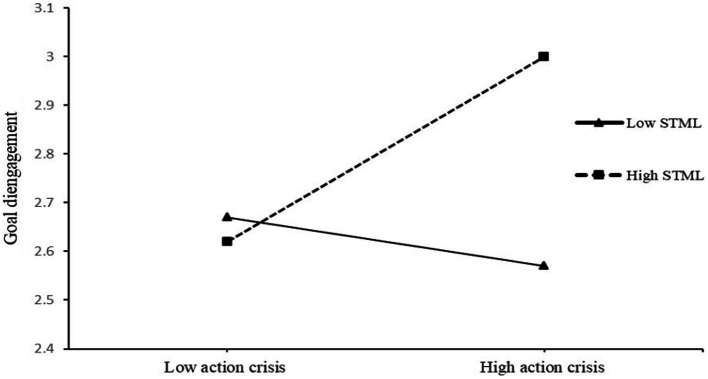
Effects of action crisis and self-transcendence meaning of life on goal disengagement in Study 1. STML, self-transcendence meaning of life.

PROCESS Model 1 (Hayes, 2013) was used to determine whether goal disengagement and reengagement were influenced by the interaction of action crisis and STML (controlling for gender and age). STML was the moderation variable, and action crisis (low versus high) was the independent variable (action crisis was coded as 0 = low action crisis and 1 = high action crisis). Two separate models were conducted for goal disengagement and goal reengagement. The model for goal disengagement was significant (*F*(5,182) = 2.38, *p* < 0.05). Action crisis did not predict goal disengagement significantly, *β* = 0.14, *t* = 1.43, *p* > 0.05, 95% bootstrap CI [−0.01, 0.36], and STML did not significantly predict goal disengagement either, *β* = 0.18, *t* = 1.89, *p* > 0.05, 95% bootstrap CI [−0.05, 0.34]. The interaction of action crisis and STML was significant, *β* = 0.44, *t* = 2.39, *p* = 0.01, 95% bootstrap CI [0.08, 0.81], Δ*R*^2^ = 0.03. A test of simple slopes was completed to probe the interaction effect at high (one standard deviation above the mean) and low (one standard deviation below the mean) levels of STML. Action crisis significantly predicted goal disengagement among participants with high STML, *β* = 0.38, *t* = 2.74, *p* < 0.01, 95% bootstrap CI [0.11, 0.65], but not among participants with low STML, *β* = −0.09, *t* = −0.65, *p* > 0.05, 95% bootstrap CI [−0.37, 0.19] (see Figure 2). Finally, the *Johnson–Neyman* (J–N) test was conducted to identify the values of STML for which the simple slope of goal disengagement regressed on action crisis was statistically significant. The region of significance included values above 3.23 for STML on the four-point scale (*p* = 0.05), which included 40.96% of the participants.

**Figure 3:**
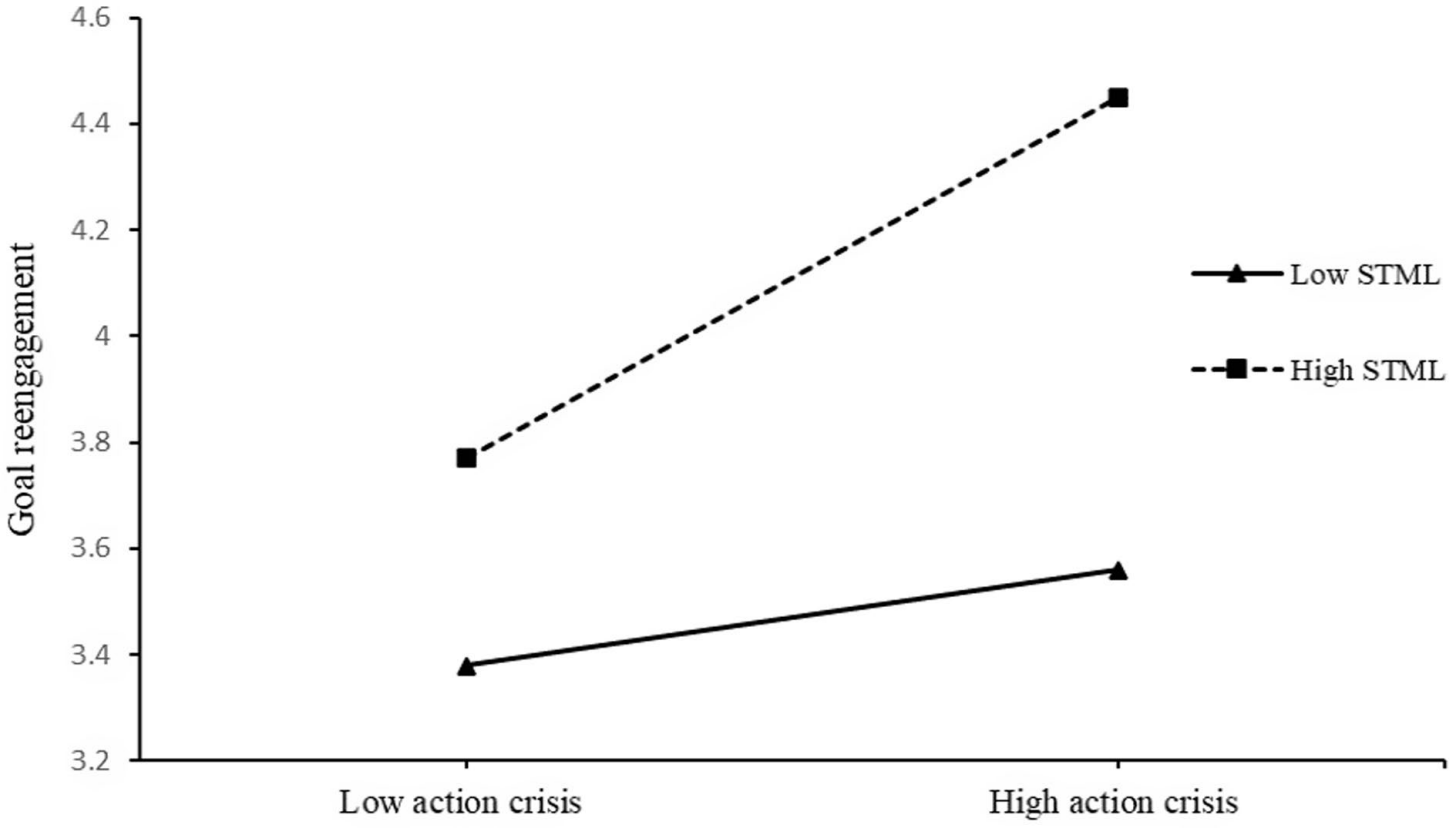
Effects of action crisis and self-transcendence meaning of life on goal reengagement in Study 1. STML, self-transcendence meaning of life.

The model for goal reengagement was also significant (*F*(5,182) = 15.85, *p* < 0.001). Both action crisis (*β* = 0.54, *t* = 4.62, *p* < 0.001, 95% bootstrap CI [0.34, 0.84]) and STML (*β* = 0.27, *t* = 3.05, *p* < 0.01, 95% bootstrap CI [0.10, 0.45]) significantly predicted goal reengagement. Furthermore, the interaction significantly predicted goal reengagement, *β* = 0.34, *t* = 2.74, *p* < 0.01, 95% bootstrap CI [0.10, 0.59], Δ*R*^2^ = 0.03. A test of simple slopes indicated that action crisis significantly predicts goal reengagement among participants with high STML, *β* = 0.67, *t* = 5.29, *p* < 0.001, 95% bootstrap CI [0.42, 0.93], but not among participants with low STML, *β* = 0.17, *t* = 0.13, *p* > 0.05, 95% bootstrap CI [−0.08, 0.44] (see Figure 3). Results of the J-N test indicated that the region of significance included values above 2.68 for STML on the four-point scale (*p* = 0.05), which included 80.32% of the participants. Therefore, higher STML promoted higher goal disengagement and reengagement among participants who read the scenario with higher action crisis.

Study 1 demonstrated that action crisis predicts goal disengagement and reengagement in participants with high STML, and the results provided support for hypothesis 1. Participants with high STML who read the high action crisis scenario were more likely to select goal adjustment choices than those who read the low action crisis scenario. Among participants with low STML, there was no difference on goal disengagement or reengagement between high and low action crisis. Furthermore, participants with high STML who read the high action crisis scenario exhibited significantly higher goal disengagement and reengagement, suggesting that STML may provide flexibility for individuals in high action crisis by promoting goal-adjusted choices.

## 4. Study 2

In Study 2, we aimed to test hypothesis 1 again using participants’ own life experiences. Furthermore, in Study 2, we also aimed to test the mediating role of perceived self-efficacy. We tested whether the effect of action crisis on goal adjustment was mediated by perceived self-efficacy, and whether the meditation was depended on STML. Based on the function of perceived self-efficacy in self-regulation (Bandura, 2012; Wolf et al., 2018), we hypothesized that perceived self-efficacy may have a positive effect on goal disengagement and reengagement. Additionally, we hypothesized that action crisis may reduce self-efficacy for participants with low STML and subsequently result in fewer goal adjustments (hypothesis 2).

### 4.1. Method

#### 4.1.1. Participants and procedure

Two hundred and forty-four undergraduate students from a university located in Beijing completed a questionnaire as a part of an elective course in exchange for course credit. All the participants were different from those in Study 1. The inclusion criteria were the same as Study 1. Finally, 223 participants [117 males, age ranged from 15 to 29, mean age = 20.38 (*SD* = 2.13)] were included in the analyses for Study 2.

Participants completed online questionnaires, the same as in Study 1. First, participants completed the STML scale. Participants were then instructed to write the most important goal that they would like to achieve within 1 year, and then estimate how much effort they had already made to achieving that goal on a 7-point scale (1 = no effort at all; 7 = a lot of effort). Next, participants completed the Action Crisis Scale (six item version), self-efficacy scale, goal disengagement scale and goal reengagement scale based on the specific goal they wrote. Finally, participants completed items concerning their demographic characteristics.

#### 4.1.2. Measures

#### 4.1.2.1. Self-transcendence meaning of life

The Self-transcendence Meaning of Life (STML) Scale was completed similar to that used in Study 1. The scale demonstrated good internal consistency in the present study (Cronbach’s *α* = 0.79 for the obtain the meaning of the failure dimension; Cronbach’s *α* = 0.83 for the detachment from success or failure dimension; Cronbach’s *α* = 0.86 for the whole scale).

##### 4.1.2.2. Action crisis

The full version of Action Crisis Scale (Brandstätter and Schüler, 2013), containing six items, was used to determine the action crisis level toward participants’ goal. Participants rated action crisis level on a 5-point scale (1 = strongly disagree, 5 = strongly agree). The scale demonstrated good internal consistency (Cronbach’s *α* = 0.82).

##### 4.1.2.3. Perceived self-efficacy

Four items were used to determine the perceived self-efficacy of participants (Vohs et al., 2013): incapable/capable, incompetent/competent, irresponsible/responsible, and lazy/hardworking. Each item listed the two opposing traits as the endpoints of a 7-point scale. Participants were asked to evaluate how they felt about themselves on the abovementioned aspects when considering the goal they wrote using a 7-point scale (1 = not at all to 7 = very much; Cronbach’s *α* = 0.94).

##### 4.1.2.4. Goal disengagement and goal reengagement

Participants completed the goal disengagement and reengagement scales similar to Study 1. In Study 2, the expressions were the same as in the original version and did not change, since Study 2 was about participants’ personal goals (e.g., “*I find it difficult to stop trying to achieve my goal*” for goal disengagement, and “*I will seek other meaningful goals*” for goal reengagement). Cronbach’s *α* was 0.71 for goal disengagement and 0.93 for goal reengagement in Study 2.

### 4.2. Results and discussion

**Figure 4:**
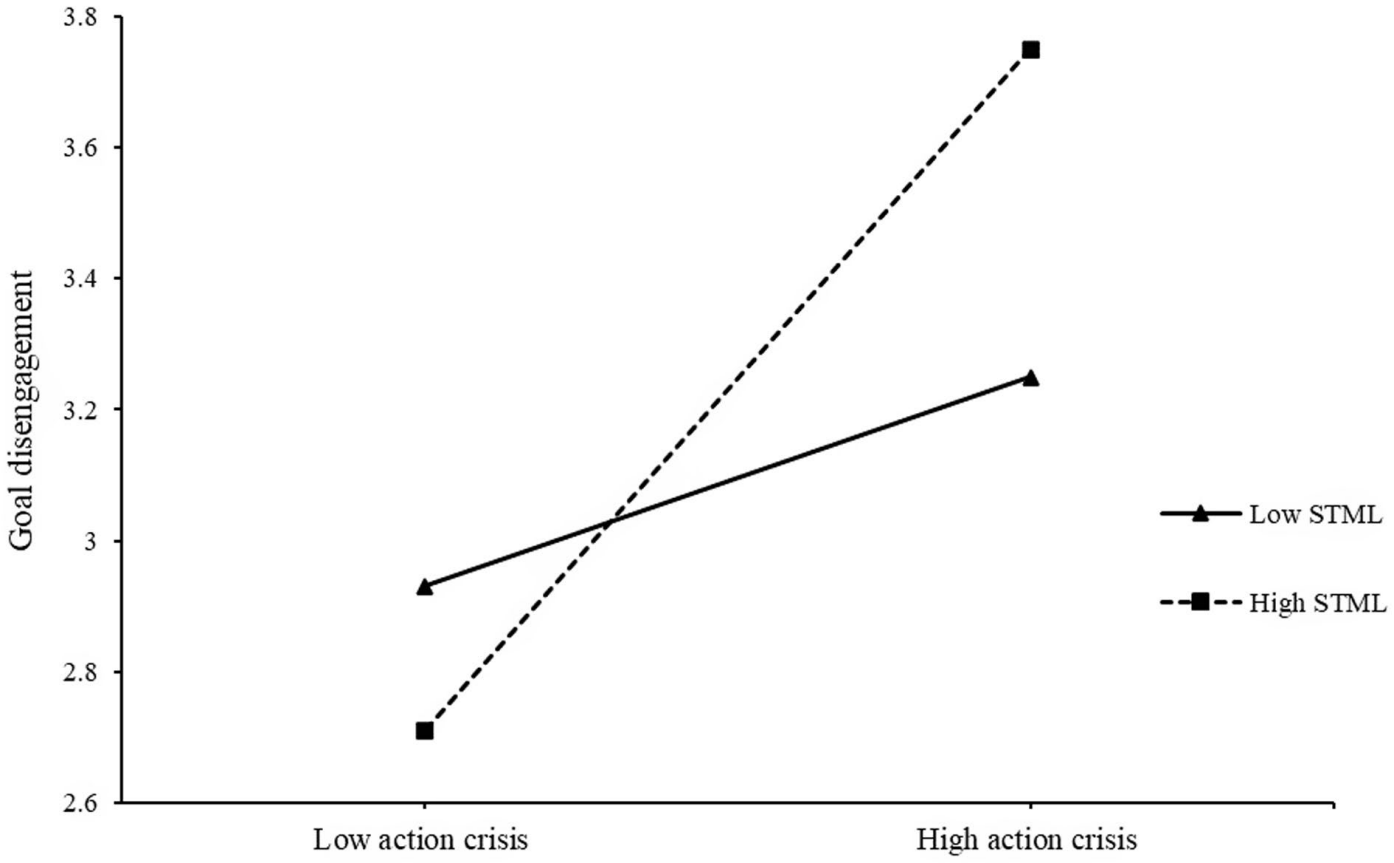
Effects of action crisis and self-transcendence meaning of life on goal disengagement in Study 2. STML, self-transcendence meaning of life.

Goal disengagement was significantly associated with goal reengagement, *r* = 0.55, *p* < 0.001, confirming findings from Study 1. The PROCESS Model 1 (Hayes, 2013) was used to examine the interaction effect of action crisis and STML on goal disengagement (controlling for gender, age and the effort level participants evaluated). This moderation model was significant, *F*(6, 216) = 24.69, *p* < 0.001. Notably, action crisis significantly predicted goal disengagement, *β* = 0.46, *t* = 7.75, *p* < 0.001, 95% bootstrap CI [0.34, 0.58], while STML did not predict goal disengagement, *β* = 0.10, *t* = 1.62, *p* > 0.05, 95% bootstrap CI [−0.02, 0.21]. The interaction between action crisis and STML was significant, *β* = 0.25, *t* = 5.59, *p* < 0.001, 95% bootstrap CI [0.16, 0.33], Δ*R*^2^ = 0.09. A test of simple slopes indicated that at both high and low levels of STML (+/− 1 SD, respectively), action crisis significantly predicted goal disengagement (high: *β* = 0.67, *t* = 11.48, *p* < 0.001, 95% bootstrap CI [0.55, 0.78]; low: *β* = 0.20, *t* = 2.48, *p* = 0.01, 95% bootstrap CI [0.04, 0.37]; see Figure 4). Results of the J-N test indicated that the region of significance included values above 2.57 for STML on the four-point scale (*p* = 0.05), which included 84.30% of the participants.

**Figure 5:**
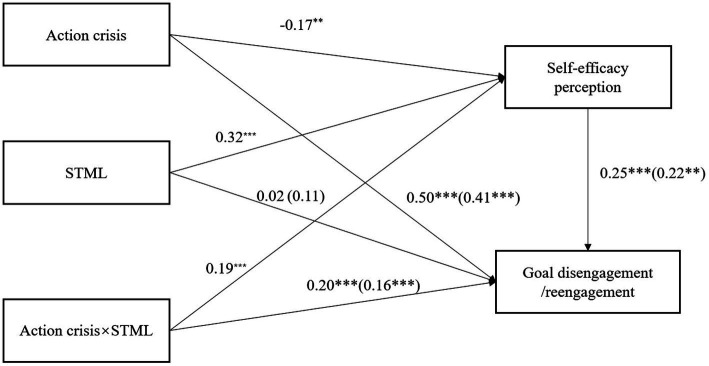
The mediation effect of perceived self-efficacy between the action crisis × STML interaction on goal disengagement and reengagement in Study 2. STML, self-transcendence meaning of life. The effects on goal reengagement are in parentheses. Some paths have no parentheses because the effects are the same for the two models. **p < 0.01; ***p < 0.001.

PROCESS Model 8 was used to perform a moderated mediation analysis to determine whether the perceived self-efficacy mediated the association between the action crisis and STML and goal disengagement. In the model, STML was entered as the moderator, and perceived self-efficacy was entered as the mediator. Action crisis significantly predicted perceived self-efficacy, *β* = −0.17, *t* = −3.15, *p* < 0.01, 95% bootstrap CI [−0.28, −0.06], as did the interaction of action crisis and STML, *β* = 0.19, *t* = 4.66, *p* < 0.001, 95% bootstrap CI [0.11, 0.27]. Perceived self-efficacy also significantly predicted goal disengagement, *β* = 0.25, *t* = 3.47, *p* < 0.001, 95% bootstrap CI [0.11, 0.39], and when perceived self-efficacy was added into the model, the interaction of action crisis and STML significantly predicted goal disengagement, *β* = 0.20 (c_3_’), *t* = 4.41, *p* < 0.001, 95% bootstrap CI [0.11, 0.29] (see Figure 5). An analysis on the conditional indirect effects demonstrated that perceived self-efficacy mediated action crisis and goal disengagement in participants with lower STML(−1SD) (indirect effect = −0.09, *se* = 0.05, 95% bootstrap CI [−0.22, −0.02]). The effect of perceived self-efficacy on goal disengagement was positive, thus, high action crisis reduced perceived self-efficacy and consequently reduced goal disengagement in participants with lower STML. For participants with higher STML(+1SD), perceived self-efficacy did not mediate the relationship between action crisis and goal disengagement (indirect effect = 0.01, *se* = 0.02, 95% bootstrap CI [−0.03, 0.04]). Hence, perceived self-efficacy partially mediated the association between action crisis and goal disengagement for participants with low STML, with an index of moderated mediation of 0.05 (c_3_ = 0.25 from the simple moderation analysis; c_3_ − c_3_’ = a_3_b_1_; in the present study we had 0.25–0.20 = 0.05), *se* = 0.03, 95% bootstrap CI [0.01, 0.12], but the mediation effect was not significant for participants with high STML.

**Figure 6:**
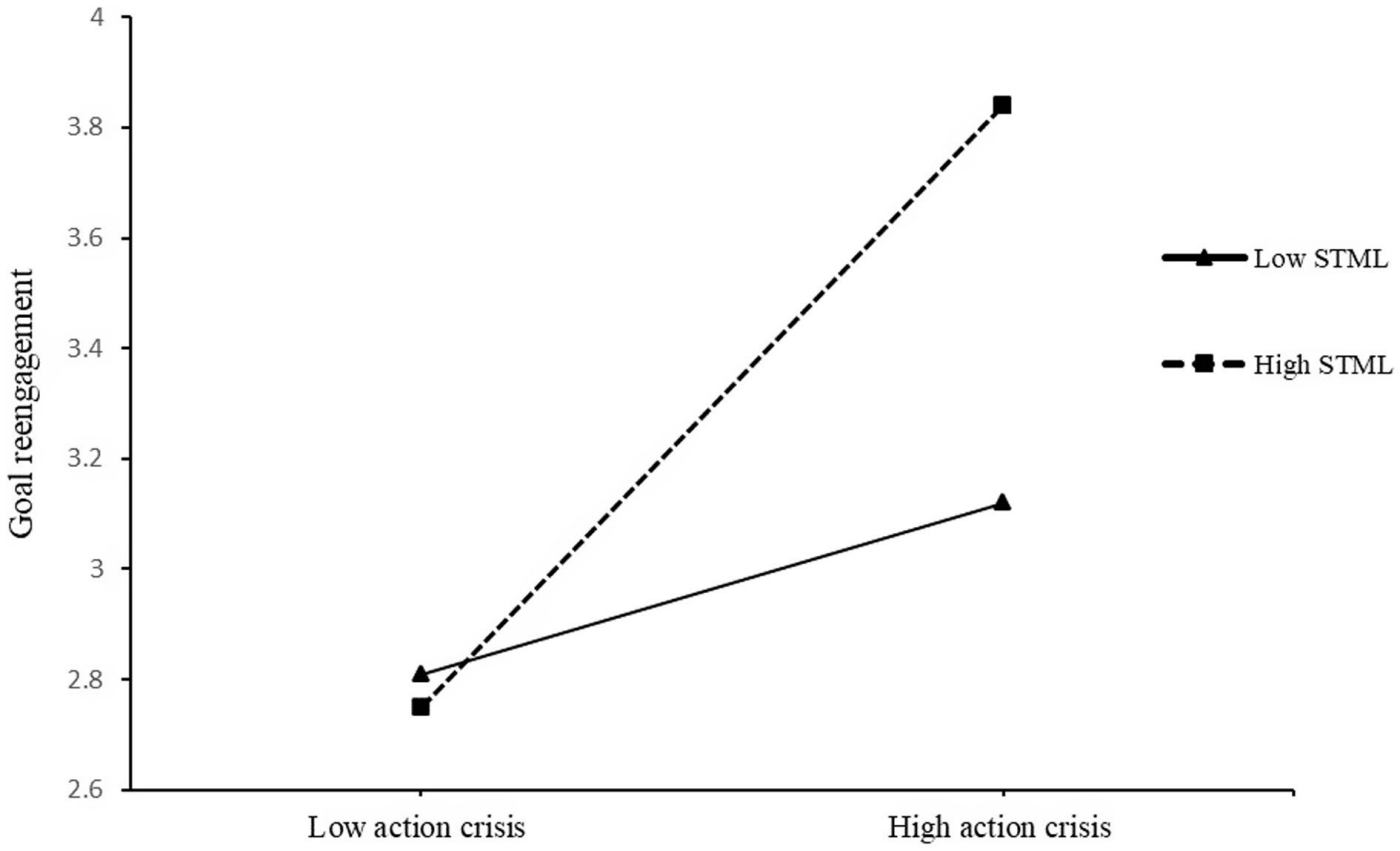
Effects of action crisis and self-transcendence meaning of life on goal reengagement in Study 2. STML, self-transcendence meaning of life.

The PROCESS Model 1 that examined the interaction between action crisis and STML on goal reengagement was also significant, *F*(6,216) = 16.16, *p* < 0.001. Specifically, action crisis significantly predicted goal reengagement, *β* = 0.37, *t* = 5.78 *p* < 0.001, 95% bootstrap CI [0.24, 0.49], and STML also significantly predicted goal reengagement, *β* = 0.18, *t* = 2.80, *p* < 0.01, 95% bootstrap CI [0.05, 0.30]. Further, the interaction between action crisis and STML significantly predicted goal reengagement, *β* = 0.20, *t* = 4.24, *p* < 0.001, 95% bootstrap CI [0.11, 0.30], Δ*R*^2^ = 0.06. A test of simple slopes indicated that action crisis significantly predicted goal reengagement at high levels of STML (+1SD; *β* = 0.69, *t* = 8.61, *p* < 0.001, 95% bootstrap CI [0.53, 0.85]), but not low levels of STML (−1SD; *β* = 0.20, *t* = 1.81, *p* > 0.05, 95% bootstrap CI [−0.02, 0.42]) (see Figure 6). The J-N test indicated that the region of significance included values above 2.66 for STML on the four-point scale (*p* = 0.05), which included 77.13% of the participants.

The PROCESS Model 8 indicated that perceived self-efficacy significantly predicted goal reengagement, *β* = 0.22, *t* = 2.87, *p* < 0.01, 95% bootstrap CI [0.07, 0.38]. When perceived self-efficacy was added into the model, the interaction also significantly predicted goal reengagement, *β* = 0.16 (c_3_’), *t* = 3.24, *p* = 0.001, 95% bootstrap CI [0.06, 0.26] (see Figure 5). A conditional mediation effects analysis revealed that perceived self-efficacy significantly mediated the association between action crisis and goal disengagement in participants with lower STML (indirect effect = −0.08, *se* = 0.04, 95% bootstrap CI [−0.18, −0.02]). Together, the negative mediation effect suggests that the effect of action crisis is more effective in reducing self-efficacy among participants with lower STML, which ultimately lowered goal reengagement. For participants with higher STML, the mediation effect was not significant (indirect effect of 0.00, *se* = 0.01, 95% bootstrap CI [−0.02, 0.04]). Thus, a partial mediation effect was also established, with an index of moderated mediation of 0.04 (c_3_ = 0.20 from the simple moderation analysis; c_3_ − c_3_’ = a_3_b_1_; in the present study we had 0.20–0.16 = 0.04), *se* = 0.02, 95% bootstrap CI [0.01, 0.10].

The results in Study 2 exhibited the same pattern with Study 1, confirming that high STML promotes goal adjustment in high action crisis. When considering their own personal goals, participants with high STML were more likely to choose to disengage or reengage the goal in high action crisis compared with low action crisis. Hypothesis 1 was supported again. Study 2 also demonstrated the moderated mediation of perceived self-efficacy. Individuals who had lower STML were more likely to be influenced by high action crisis, reflective of lowered perceived self-efficacy. Moreover, the study found that perceived self-efficacy had a positive effect on goal adjustment. Action crisis had a negative effect on self-efficacy for individuals with low STML, and this process was subsequently associated with goal adjustment. The results of Study 2 provided evidence for hypothesis 2. These results may provide novel information for examining self-conception in goal adjustment in future.

## 5. General discussion

High action crisis plays an important role in goal disengagement and represents individuals’ difficulties in pursuing goals. Notably, individuals have reported considering the benefits and costs of disengaging goals (Brandstätter and Schüler, 2013), indicating that goal disengagement may be a frequent intention. Thus, the capacity to actively disengage or reengage goals is necessary during high action crisis. Both studies completed in this manuscript suggest that STML increases goal adjustment during a high action crisis. Personal goals, especially achievement goals, represent self-image and competence, and together contribute to self-centeredness (Elliot and Hulleman, 2017). In the present study, STML referred to the attitude of reducing or removing self-centeredness (Li et al., 2019), emphasized a particular wisdom, and was related to abnegation and dialectic. Recent reports suggest that transcendence-focus relieves preoccupation with threat situations and helps individuals who are attempting to remove burdens feel more relaxed (McGregor et al., 2022). Self-transcendence has also been associated with positive outcomes like life purpose, resilience, and mental health (Nygren et al., 2005). Our findings add to this breadth of literature in goal adjustment and self-regulation. In the frame of STML, individuals in high crisis may choose to forego unattainable goals rather than give them up or ineffectively persist in the pursuit of a goal. The present study indicates that some transcendent belief is beneficial to self-regulation, particularly in situations in which goal adjustment may be necessary.

Mechanistically, STML appears to work predominately through perceived self-efficacy. Prior studies have demonstrated that self-efficacy is an important factor in goal management and self-regulation (Judge et al., 2007). However, there were two apparent conflicting aspects within the effect of self-efficacy in prior studies (Gielnik et al., 2020). One aspect focused on the persistence of self-efficacy (Wright et al., 2012). For example, as the difficulty of the task increased, individuals with higher self-efficacy tended to persist more than individuals with lower self-efficacy (Beattie and Davies, 2010). Vohs et al. (2013) also found that decreases in self-efficacy could promote goal disengagement. The second aspect focused on the resilience of self-efficacy (Bandura, 1997). In contrast to Vohs et al. (2013), results from the present study suggest a positive relationship between higher self-efficacy and goal disengagement and reengagement, both of which support the second aspect of the self-efficacy effect. Here, we suggest that self-efficacy positively relates to adjustment, as perceived self-efficacy resulted in higher malleable resilience (Bandura, 1997; Pajares and Schunk, 2001), which may lead to better coping abilities in individuals struggling with high action crisis (Benight and Cieslak, 2011). Recently, self-efficacy has been recognized as an important factor mediating stress responses (Lannin et al., 2018). University students with more flexible coping strategies (i.e., positive reappraisal and planning) exhibited significantly higher self-efficacy (Freire et al., 2020). Flexible goal adjustment and self-efficacy was also positively correlated in patients with brain injuries (Brands et al., 2014). Notably, Brands et al. (2014) used disengagement from blocked goals, finding a new orientation, and acceptance to the constrained situation to measure flexible goal adjustment. Several hypotheses have also suggested that individuals with high self-efficacy may feel less conflicted when encountering challenges because they believe they are competent in different situations (Chen et al., 2001). Thus, when self-efficacy cannot sustain effort toward a goal, it can bring flexibility allowing individuals to take adaptive action. This concept becomes important when individuals experience high action crises, as it helps one rebuild and restore the concept of “self” when disengaging from previous self-conceptualizing goals. Thus, participants with higher perceived self-efficacy often have more perceived courage to disengage from goals due to positive self-perception. The flexibility of self-efficacy was also supported by the positive correlation with goal reengagement (Study 2). Results revealed that participants with higher self-efficacy were ready to choose and commit to new goals, indicating higher confidence and forward-thinking (Wu et al., 2021).

A moderated mediation effect (Study 2) was demonstrated among action crisis, self-efficacy, STML, and goal disengagement/reengagement. Specifically, action crisis was not associated with perceived self-efficacy of participants with higher STML. Hence, higher STML may help individuals remain stable in high-stress situations. However, it appeared that self-efficacy was influenced by action crisis in participants with lower STML. Among participants with lower STML, the action crisis could negatively influence perceived self-efficacy significantly, and then negatively influence goal adjustment. The concept of STML includes understanding that gain and loss is inevitable and meaningful, and believing in the dialectical relationship between success and failure (Li, 2006). The present study revealed self-transcendence could contribute to participants’ acceptance of blocked situations and build a suitable understanding of gain and loss, which could protect self-efficacy from being influenced by action crisis, and further promote goal adjustment.

It is notable that the same influential pattern of action crisis and STML was found on goal disengagement and reengagement in the present study. Wrosch et al. (2003) similarly demonstrated that people with high goal disengagement and reengagement exhibited higher well-being. Indeed, only those who disengage from unattainable goals without reengaging new goals report feeling lonely and empty (Scheier and Carver, 2003). Thus, the ability to pursue new goals is arguably equally as important as goal disengagement. Considering future options and reframing one’s perspective may help individuals become motivated to disengage from unattainable goals, particularly during high action crisis. Future studies may focus on the influence, sequence and interaction of goal disengagement and reengagement with mental health.

### 5.1. Implications of the present study

Although research on goal disengagement and reengagement has recently become more prevalent, several domains, details, and mechanism about goal adjustment remain unknown. Many influential factors about goal reengagement remain unclear, and there are few empirical studies on the relationship between self-concept and goal-regulation strategies (Neal et al., 2017). The present study focused on the above issues and described relationships among meaning of life, self-efficacy, and goal adjustment. Furthermore, we demonstrated the influence of STML on goal adjustment, and suggest that Eastern-philosophy wisdom may be beneficial during goal disengagement and reengagement processes, which could promote better mental health.

### 5.2. Limitations and future directions

This study was limited by our measurement of goal disengagement and reengagement using a questionnaire rather than behavioral domains. Despite focusing on the capability of goal adjustment in different level of action crisis, we used a self-report scale to measure the two variables similar to prior work (Wrosch et al., 2003; Haase et al., 2021). We also used hypothetical situations to represent life domains in Study 1, which does not provide information about how individuals may react when encountering an action crisis in their personal lives. Future studies should document the causal relationship between STML, action crisis, and actual goal disengagement and reengagement behavior. Furthermore, in the present study, we used the personal trait evaluation to measure self-efficacy. We did not use published scales to measure self-efficacy in the present study because we intended to examine perceived self-efficacy according to participants’ personal goals and action crisis situations rather than examining self-efficacy as a general trait characteristic. Examining personal traits that aligned with personal situations was more suitable to the goals of the present study. However, self-efficacy may reflect participants’ beliefs about their capability and ability to achieve desired outcomes, potentially contributing to discrepancies in the results. It also should be noted that there are several definitions for *meaning of life* (King and Hicks, 2021). Due to the importance of meaning in life in goal engagement and self-regulation, other researchers may seek to expand these findings using other definitions of meaning of life to further elucidate the influence of meaning of life on goal adjustment. Finally, since this study was conducted in China, we cannot say whether the findings can be generalized to different cultures. Previous studies have found that self-transcendence is an important factor through which mindfulness works (Vago and Silbersweig, 2012). Studies have also indicated that self-transcendence can be understood as a particular type of wisdom in Western contexts (Reischer et al., 2021). We can infer, therefore, that STML could also be beneficial in more individualist cultures. Cross-cultural research is needed to test whether STML can promote goal adjustment under action crises in individualist cultures.

In conclusion, the present study examined the influence of STML on goal adjustment at different levels of action crisis and explored the benefits of meaning of life on goal-regulation processes. Findings from the present study describe the wisdom, meaning, and core values that may be helpful to goal disengagement and reengagement. Finally, findings from the present study further describe the mediation effects of self-efficacy among participants with lower STML, which indicates that self-efficacy plays a flexible role in goal setting and regulating processes.

## Data availability statement

The raw data supporting the conclusions of this article will be made available by the authors, without undue reservation.

## Ethics statement

Ethical review and approval was not required for the study on human participants in accordance with the local legislation and institutional requirements. Written informed consent from the participants’ legal guardian/next of kin was not required to participate in this study in accordance with the national legislation and the institutional requirements. The study followed the Declaration of Helsinki and participants’ rights were preserved and their participation was treated appropriately.

## Author contributions

HZ contributed to supervision and the validation of the study. XH contributed to study design, data analysis, and original manuscript. MG contributed to data collection and reviewed the manuscript. All authors contributed to the article and approved the submitted version.

## Funding

This study was supported by Ministry of Education, Humanities and Social Sciences Project (22YJA190004), and Ministry of Education Youth Project, National Education Sciences Planning of China (EBA220551).

## Conflict of interest

The authors declare that the research was conducted in the absence of any commercial or financial relationships that could be construed as a potential conflict of interest.

## Publisher’s note

All claims expressed in this article are solely those of the authors and do not necessarily represent those of their affiliated organizations, or those of the publisher, the editors and the reviewers. Any product that may be evaluated in this article, or claim that may be made by its manufacturer, is not guaranteed or endorsed by the publisher.
